# Acute Effects of a Moderate Static Magnetic Field on Gypsy Moth (*Lymantria dispar*) Larvae †

**DOI:** 10.3390/insects17040402

**Published:** 2026-04-08

**Authors:** Dajana Todorović, Marija Mrdaković, Larisa Ilijin, Milena Vlahović, Milena Janković-Tomanić, Dragana Matić, Aleksandra Filipović, Vesna Perić-Mataruga

**Affiliations:** Department of Insect Physiology and Biochemistry, Institute for Biological Research “Siniša Stanković”, National Institute of the Republic of Serbia, University of Belgrade, Despot Stefan Blvd. 142, 11108 Belgrade, Serbia; mm1507@ibiss.bg.ac.rs (M.M.); lararid@ibiss.bg.ac.rs (L.I.); minavl@ibiss.bg.ac.rs (M.V.); miljan@ibiss.bg.ac.rs (M.J.-T.); dragana.matic@ibiss.bg.ac.rs (D.M.); aleksandra.mrkonja@ibiss.bg.ac.rs (A.F.); vesper@ibiss.bg.ac.rs (V.P.-M.)

**Keywords:** *Lymantria dispar*, magnetic field, preadult development, mortality, instar duration, antioxidant defence

## Abstract

Magnetic fields are abiotic environmental factors that can cause a wide range of biological effects at both the cellular and whole-organism levels. In this study, we investigated the effects of a static magnetic field (SMF, 110 mT) on life history traits and antioxidant defence mechanisms during the preadult development of *Lymantria dispar*. SMF exposure did not affect the mass of younger larvae, whereas older larvae and pupae showed significantly reduced mass compared to controls. Estimated larval mortality was higher in the SMF group, while developmental duration was significantly prolonged in the fifth larval instar and in both male and female pupae. SMF induced stage-dependent modifications in antioxidant defence. Superoxide dismutase activity and catalase activities were significantly increased, predominantly in later developmental stages, while glutathione reductase and glutathione S-transferase showed instar-dependent responses. In addition, the content of total and oxidised glutathione was significantly higher in the fifth and sixth instars of SMF-exposed larvae compared to controls. The study shows that static magnetic field exposure can interfere with normal developmental processes and redox homeostasis in insects, implying potential adaptive mechanisms under stressful conditions.

## Simple Summary

Magnetic fields are environmental factors that may influence biological processes in insects. However, their effects on insect development and physiology are not yet fully understood. In this study, we investigated how exposure to a static magnetic field (110 mT) affects development and antioxidant defence in the gypsy moth, *Lymantria dispar*, an important forest pest species. Magnetic field exposure did not affect the body mass of early larval stages, but older larvae and pupae showed reduced mass compared to control individuals. Larvae exposed to static magnetic field also exhibited higher mortality, and developmental duration was significantly prolonged in the fifth larval instar and in pupae. Furthermore, magnetic field exposure altered antioxidant defence mechanisms in a stage-specific manner, particularly during later developmental stages, indicating physiological stress. A moderate static magnetic field affected larval growth and antioxidant defences in gypsy moths, highlighting how insects respond to environmental stress.

## 1. Introduction

Insect species are sensitive to magnetic fields (MFs) in their environment, partly due to their ability for magnetoreception, and they respond to changes in the intensity and characteristics of these fields. This sensitivity manifests in alterations across nearly all life processes, including metabolism, development, reproduction, fertility, and homeostasis [1,2,3].

In the course of evolution, insects—like other organisms—have been continuously exposed the Earth’s magnetic field, a natural component of their environment [4], which today represents a very important abiotic stress factor [5]. Accelerated industrial and technological development has introduced numerous new sources of exposure to magnetic fields with different characteristics [6]. As these magnetic fields are several times stronger than the Earth’s field, they may elicit responses at all levels of biological organization [7]. Static magnetic fields (SMFs) are time-independent fields of constant strength [8], and are categorised as weak, moderate, strong, or ultrastrong [9]. The biological effects caused by the action of SMF depend on several parameters, including intensity, homogeneity and exposure time, as well as on the different biological samples analysed [10]. Magnetic fields are known to modify the electromagnetic properties of biomolecules, alter membrane permeability and ion flux, modulate enzyme activity in biochemical reactions, and affect the concentration and activity of reactive oxygen species (ROS), as well as cellular growth and differentiation [11,12].

One of the most important mechanisms by which magnetic fields interact with biological systems is the mechanism of radical pairs. Specifically, magnetic fields disturb the interconversion between the singlet and triplet states of free radical molecules in tissues [13]. These molecules, which contain one or more unpaired electrons, and thus, possess high reactivity, act as magnetosensitive agents and are involved in numerous biochemical processes [12]. The most common free radicals are oxygen- or nitrogen-based compounds with unpaired electron(s), commonly referred to as reactive oxygen species (ROS), which predominate in biological systems, and reactive nitrogen [14].

Superoxide anion (O2^•−^), hydroxyl radical (^•^OH), and hydrogen peroxide (H_2_O_2_) are the most physiologically significant free radicals [15]. ROS can be classified as either free radicals or non-radicals. These species are continuously produced as by-products of normal cellular metabolism and, in low to moderate concentrations, play essential roles in various cellular functions and biological processes, being involved in physiological cellular activities [16]. At high concentrations, when there is a pronounced imbalance between the production and removal of reactive oxygen species, oxidative stress occurs—defined as an imbalance between the generation of free radicals and the antioxidant defences. The production of free radicals is regulated by antioxidant defence mechanisms, which consist of enzymatic and non-enzymatic components that remove or block the harmful effects of ROS and protect cellular components from oxidative damage by acting as “free radical scavengers” [16,17].

The antioxidative defence system in insects primarily consists of enzymes such as superoxide dismutase (SOD), which catalyses the dismutation of the superoxide anion radical; catalase (CAT), which decomposes hydrogen peroxide into water and hydrogen; glutathione reductase (GR), which converts oxidised glutathione into its active, reduced form (though it does not directly eliminate ROS); ascorbate peroxidase (APOX), which catalyses the reduction of hydrogen peroxide to water; and the phase II biotransformation enzyme glutathione-S-transferase (GST), which catalyses the conjugation of electrophilic toxic molecules to glutathione. In addition to these enzymatic antioxidants, non-enzymatic cellular antioxidants also contribute to defence, including glutathione (GSH), which maintains redox homeostasis, ascorbic acid, carotenoids, and α-tocopherol, as well as water-soluble molecules (e.g., uric acid, carbohydrates, polyols) and iron-binding proteins such as ferritin and transferrin [18,19]. Antioxidant defences are believed to play a key role in supporting normal development. In insects, survival, development, growth, and lifespan are influenced by the regulation of the balance between free radicals and antioxidants [20]. However, there are limited data on the activity of the antioxidant defence system across different stages of preadult insect development.

The model system in our research is the insect *Lymantria dispar* (Linnaeus, 1758), Lepidoptera. It is well known that insects, like other organisms, are sensitive to the effects of various environmental stressors, including magnetic fields [21,22]. Therefore, the aim of this study was to investigate the acute effect (48 h) of a static magnetic field (SMF; 110 mT) on fitness-related traits (development time, mass, and mortality), as well as on the antioxidant defence system—specifically the activities of SOD, CAT, GR, and GST, and the content of total GSH (GSHt) and oxidised glutathione (GSSG)—in different larval instars (first–third: younger larvae; fourth–sixth: older larvae) and in pupae of the gypsy moth *L. dispar*. This approach provides an opportunity to assess whether the selected parameters are associated with SMF-induced stress and to identify which phase of preadult development is most sensitive (i.e., with potential as a bioindicator) to the presence of this environmental stressor.

## 2. Materials and Methods

### 2.1. Model Organisms

The experimental model used in this study was the gypsy moth, *L. dispar*. This holometabolous, polyphagous herbivore is a known pest in forest ecosystems, orchards, and urban parks throughout the Northern Hemisphere. Its development proceeds through 6 larval instars—5 in males and 6 in females. Due to its relatively short development time, ease of manipulation under laboratory conditions, and high sensitivity to various environmental changes (e.g., heavy metals, temperature fluctuations, magnetic fields), the gypsy moth is considered a suitable experimental model and bioindicator species [23].

For this study, gypsy moth egg masses were collected from an oak forest near Bačka Palanka (Vojvodina, Serbia) and stored in a refrigerator (at 4 °C) until hatching. Hatching occurred under controlled laboratory conditions: temperature of 23 ± 0.5 °C, light intensity 1000 lx, relative humidity 75%, and a 12 h light/12 h dark photoperiod. Egg masses were cleaned of hairs and disinfected in 0.1% sodium hypochlorite for 5 min, then rinsed with distilled water and air-dried—an essential step to prevent contamination by pathogens. After hatching, larvae were fed an artificial diet optimised for gypsy moth rearing [24]. Larval moulting was monitored daily.

### 2.2. Experimental Procedure

Immediately after hatching, *L. dispar* larvae were randomly divided into two experimental groups: a control group and a group exposed to a static magnetic field (SMF, 110 mT). Larvae were kept in Petri dishes (radius = 3.5 cm). During the first and second instars, 10 larvae were placed in each dish, while from the third to the sixth instar, larvae were reared individually.

Because of the limited size of the magnet, exposure was conducted in four consecutive experimental runs. For the mortality and developmental monitoring experiment, 30 larvae per treatment group were initially used. Individuals were monitored daily throughout larval development, and mortality was recorded each day; therefore, the number of surviving larvae decreased progressively across instars. Biochemical analyses were performed independently in three biological batches, each starting with 150 larvae per treatment group. Body mass was measured prior to biochemical processing, and the same individuals were then used for analyses. Measurements were taken on the third day after moulting into each new instar, at which point larvae (or pupae) were sacrificed. Larvae were weighed individually, except for first- and second-instar larvae, which—due to their small size—were weighed in groups of 10 to minimise measurement error.

### 2.3. Magnetic Field

The magnetic field was generated by a double U-shaped permanent magnet (Model 6002, Raytheon, Waltham, MA, USA). Experimental individuals (larvae and pupae of *L. dispar*) were exposed to a SMF for 48 h from the first day after moulting into a new developmental stage. This period coincides with a phase of intensified physiological activity following entry into a new larval instar. The average magnetic flux density was 110 mT, with a field homogeneity of ±26.87%. The applied SMF is within the range commonly used in experimental studies on various biological systems, has been shown to exert effects at different levels of biological organisation, and is close to the values used for diagnostic purposes [25]. The permanent magnet and the distribution of the magnetic field are described in detail in our previous publication [26]. The magnetic field lines were oriented parallel to the vertical component of the geomagnetic field. The magnetic field was measured using a GM05 gaussmeter equipped with a PT 2837 probe (Hirst Magnetic Instruments, Falmouth, UK). During the experiment, the ambient magnetic field in the experimental room was within the normal range (~47 μT). The average local geomagnetic field (44° 38′ N, 20° 46′ E, measured by GSM-19 v6.0 proton magnetometer: GEM SYSTEMS INC, Markham, ON, Canada) ranged from 47,500 to 47,532 nT for the vertical component, while the horizontal component varied between 22,643 and 22,660 nT (Republic Geodetic Authority, Belgrade, Republic of Serbia). The background magnetic field did not exceed 10^−5^ mT.

### 2.4. Preparation of Homogenates and Enzymatic Assays

#### 2.4.1. Preparation of Homogenates

Homogenates were prepared from whole larvae of *L. dispar*, with 2–15 individuals pooled per homogenate depending on larval mass (10–15 for first and second instars, 3–4 for third and fourth instars, and 2–3 for fifth and sixth instars). Each pooled homogenate represented one biological replicate. Eight biological replicates (pools of *L. dispar* larvae) per treatment and developmental stage were used for enzymatic assays. On the third day after moulting into a new larval instar, all individuals were euthanised using liquid nitrogen. Larvae were homogenised on ice in 0.25 M sucrose buffer (pH 7.4) using an Ultra-Turrax homogeniser (IKA-Werke, Staufen, Germany) in three cycles of 10 s with 15 s pauses at 2000 rpm [26]. The homogenates were then sonicated using a Sonoplus HD2070 instrument (Bandelin, Berlin, Germany) in three cycles of 10 s with 10 s pauses [27]. To a portion of the sonicated homogenate, 5% sulfosalicylic acid (Sigma-Aldrich Chemie, Steinheim, Germany) was added in a 2:1 ratio. GSH content was measured after centrifugation for 20 min at 10,000× *g* and 4 °C (Model 5417R, Eppendorf, Hamburg, Germany). The remaining homogenates were centrifuged using a Beckman L7-55 ultracentrifuge (Beckman, Nyon, Switzerland) at 37,500 rpm for 90 min at 4 °C. The resulting supernatants were stored at −20 °C until further assays.

#### 2.4.2. Enzymatic Activity Assays

Total protein concentration in all samples was quantified by the Bradford method [27], using bovine serum albumin as the standard.

SOD activity was assessed using the epinephrine method, which is based on the ability of SOD to inhibit the autoxidation of epinephrine (adrenaline) to adrenochrome in an alkaline medium [28]. This reaction releases superoxide anion radicals, which accelerate the autoxidation of adrenaline. The rate of this autoxidation was determined spectrophotometrically at 25 °C by monitoring absorbance at a wavelength of 480 nm. One unit of SOD activity was defined as the amount of enzyme required to cause 50% inhibition of the autooxidation of adrenaline and was expressed as specific activity (U/mg protein).

CAT activity was determined according to the method of Beutler [29], by measuring decomposition of the substrate hydrogen peroxide (10 mM H_2_O_2_) at 230 nm over a period of 3 min.

For GR activity we used NADPH and oxidised glutathione (GSSG) as substrates, based on the ability of GR to catalyse the reduction in GSSG to reduced glutathione (GSH) [30]. The activity of this enzyme was determined at 340 nm.

GST activity was measured at 340 nm and 37 °C using the method of Habig [31]. GSH and 1-chloro-2,4-dinitrobenzene (CDNB) were used as substrates. This method is based on the reaction of CDNB with the -SH group of GSH, catalysed by GST present in the samples.

GSHt content was determined according to Griffith [32]. This method is based on a recycling process in which GSH is successively oxidized by 5,5-dithiobis-2-nitrobenzoic acid (DTNB) and reduced by NADPH. The rate of formation of 2-nitro-5-thiobenzoic acid was monitored at 412 nm, and the concentration of total glutathione was calculated in accordance with the standard.

The content of GSSG was determined at 412 nm using the method of Griffith [32], which employs vinylpyridine to mask reduced glutathione and triethanolamine to maintain the optimal pH value.

All antioxidant enzyme activities and glutathione contents were measured spectrophotometrically using a UV-160 spectrophotometer (Shimadzu Corporation, Kyoto, Japan) equipped with a temperature-controlled cuvette holder. All assays were performed in eight biological replicates per treatment and developmental stage, with each sample analysed in duplicate.

### 2.5. Statistical Analyses

Data normality and homogeneity of variances were assessed using the Shapiro–Wilk and Levene’s tests, respectively. Larval and pupal mass, as well as larval developmental duration, were analysed using General Linear Models (GLMs) with Type III sums of squares. When normality assumptions were not met, pupal developmental duration was analysed using a GLM based on ranked data (Scheirer–Ray–Hare approach). For larval and pupal life history traits, treatment, developmental stage, and sex (where applicable) were included as fixed factors. Significant effects were further examined using Bonferroni-adjusted post hoc tests. Larval mortality was analysed using a generalised linear model (GLM) with binomial error distribution and logit link function, with treatment and larval stage included as fixed factors. Effect size was expressed as odds ratios (ORs) with 95% confidence intervals. Antioxidant enzyme activities (SOD, CAT, GR, and GST), as well as total and oxidised glutathione levels (GSHt and GSSG), were analysed using two-way analysis of variance (ANOVA), with treatment and larval instar as fixed factors. When significant main effects or interactions were detected, Bonferroni-adjusted multiple comparisons were applied. Differences were considered statistically significant at *p* < 0.05. All statistical analyses were conducted using Statistica 12 (StatSoft Inc., Tulsa, OK, USA) and R 4.5.2. statistical software (R Core Team, Vienna, Austria).

## 3. Results

### 3.1. Life History Traits of L. dispar Larvae and Pupae

Larval mass was significantly affected by treatment (GLM: F_1,276_ = 434.09, *p* < 0.001), instar (F_5,276_ = 15,440.99, *p* < 0.001), and their interaction (F_5,276_ = 129.72, *p* < 0.001). Post hoc Bonferroni comparisons showed no significant differences between treatments during the first three instars (*p* > 0.05). However, from the fourth instar onwards, larvae exposed to SMF had significantly lower body mass than controls (*p* < 0.001; Table 1).

**Figure 1:**
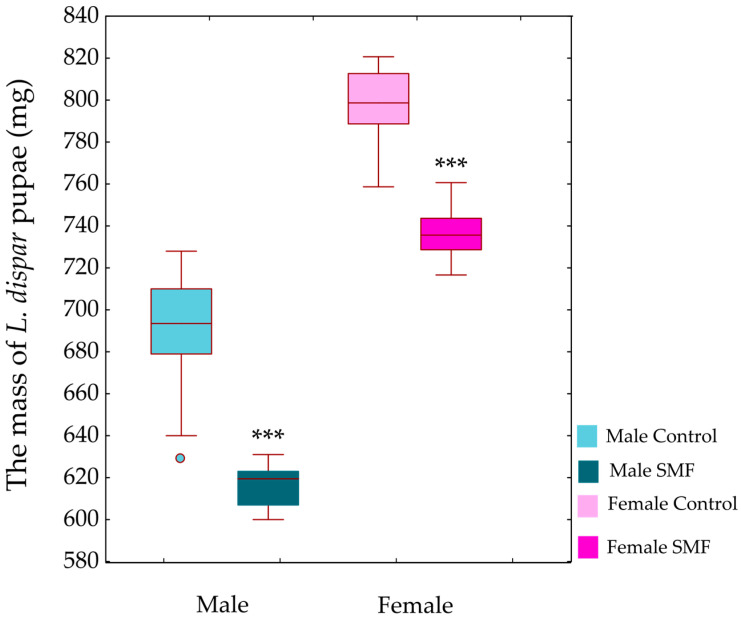
Mass of *L. dispar* pupae (mg). Data are presented as boxplots (median, first and third quartiles, minimum and maximum values; *n* = 30 in each group). Asterisks indicate significant differences compared to the control group (*** *p* < 0.001; GLM followed by Bonferroni post hoc test).

Pupal mass was significantly affected by treatment (GLM: F_1,116_ = 439.7, *p* < 0.001) and sex (F_1,116_ = 1306.0, *p* < 0.001), with a significant treatment × sex interaction (F_1,116_ = 4.1, *p* < 0.05). SMF treatment led to a significant reduction in pupal mass in both males and females compared to controls (*p* < 0.001; Figure 1).

**Figure 2:**
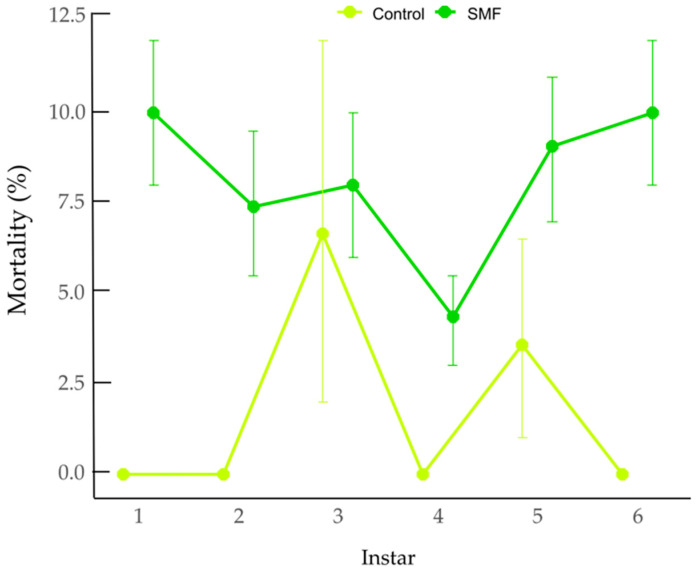
Larval mortality (%) across developmental instars in the control and SMF group (generalized linear model, binomial distribution, logit link). Error bars indicate 95% confidence intervals.

Larval mortality was significantly affected by treatment (GLM with binomial distribution and logit link), with higher mortality observed in the SMF group than in the control group (z = −2.02, *p* = 0.0437). Estimated mortality was 2.01% (95% CI: 0.62–6.33%) in the control group and 7.49% (95% CI: 4.08–13.34%) in the SMF group. The odds of mortality were significantly higher in the SMF group compared to the control (odds ratio (OR) = 3.95, 95% CI: 1.14–18.21). Larval stage did not significantly influence mortality (χ^2^ = 2.41, df = 5, *p* = 0.790), indicating comparable susceptibility across developmental instars. As shown in Figure 2, predicted mortality remained consistently higher under SMF treatment across all larval instars, whereas mortality in the control group remained low throughout development.

**Figure 3:**
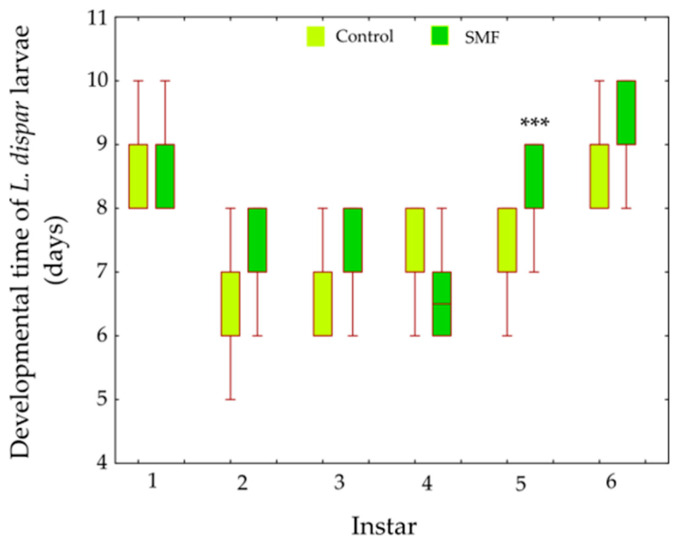
Effect of SMF (110 mT) on the development of *L. dispar* larvae. Data are shown as boxplots (median, first and third quartiles, minimum and maximum; *n* = 17–30, depending of instar). *** *p* < 0.05 indicates significant differences compared with the control group (GLM, Type III sums of squares; Scheirer–Ray–Hare approach followed by Bonferroni post hoc test).

A General Linear Model (Type III sums of squares; Scheirer–Ray–Hare approach) revealed a significant main effect of treatment (F_1,290_ = 35.68, *p* < 0.001) and instar (F_5,290_ = 94.96, *p* < 0.001) on larval development time. Importantly, a significant treatment × instar interaction was detected (F_5,290_ = 6.45, *p* < 0.001), indicating that the effect of magnetic field varied among developmental stages. Post hoc comparisons (Bonferroni-adjusted) showed that magnetic field significantly prolonged developmental duration in the fifth instar (*p* < 0.001), whereas no statistically significant differences were observed in the other larval instars (Figure 3).

**Figure 4:**
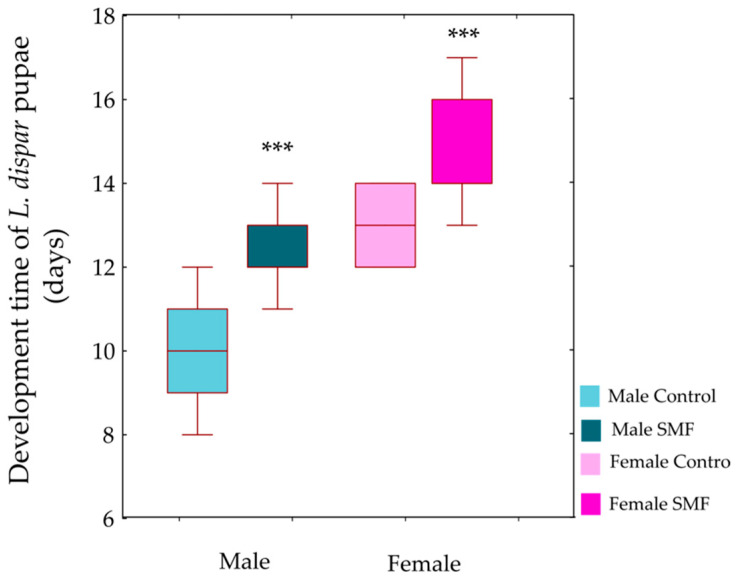
Effect of SMF (110 mT) on the development of *L. dispar* pupae. Data are shown as boxplots (median, first and third quartiles, minimum and maximum, *n* = 15 in each group). *** *p* < 0.001 indicates significant differences compared to the control group (GLM, Type III sums of squares, followed by Bonferroni post hoc test).

GLM (Type III sums of squares) revealed significant main effects of treatment (F_1,56_ = 48.67, *p* < 0.001) and sex (F_1,56_ = 79.02, *p* < 0.001) on pupal development time. The treatment × sex interaction was not significant (F_1,56_ = 0.37, *p* = 0.544), indicating that the effect of the magnetic field was consistent across sexes. Post hoc Bonferroni tests, conducted to further examine the significant main effects, confirmed that individuals exposed to the magnetic field had prolonged pupal development compared to controls in both sexes (*p* < 0.001, Figure 4).

### 3.2. The Antioxidant Defence System in Larvae of the Gypsy Moth L. dispar

**Figure 5:**
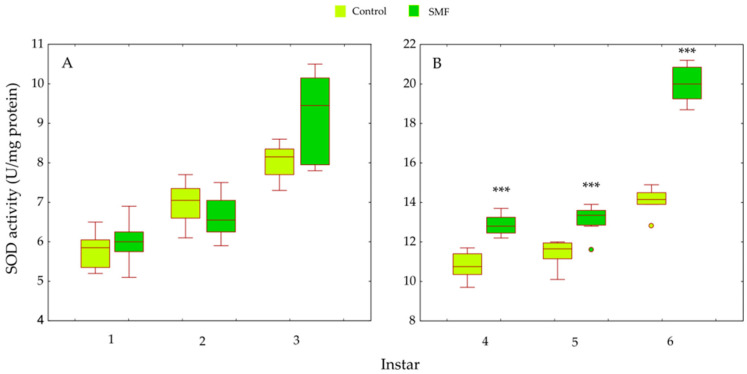
Effect of SMF (110 mT) on superoxide dismutase (SOD) activity in *L. dispar* larvae across developmental instars: (**A**) first to third instars; (**B**) fourth to sixth instars. Data are presented as boxplots (median, first and third quartiles, minimum and maximum values; *n* = 8 per group). Asterisks indicate significant differences compared to the control group (*** *p* < 0.001; two-way ANOVA followed by Bonferroni post hoc test).

Two-way ANOVA revealed significant main effects of treatment (F_1,84_ = 168.38, *p* < 0.001) and larval instar (F_5,84_ = 611.59, *p* < 0.001) on SOD activity. A significant treatment × instar interaction was detected (F_5,84_ = 43.30, *p* < 0.001), indicating an instar-dependent response. Although SOD activity tended to be higher in SMF-exposed larvae during the early instars, these differences were not statistically significant (*p* > 0.05; Figure 5A). In contrast, in older larvae (fourth to sixth instars), SMF induced a pronounced and statistically significant increase in SOD activity (*p* < 0.001; Figure 5B).

**Figure 6:**
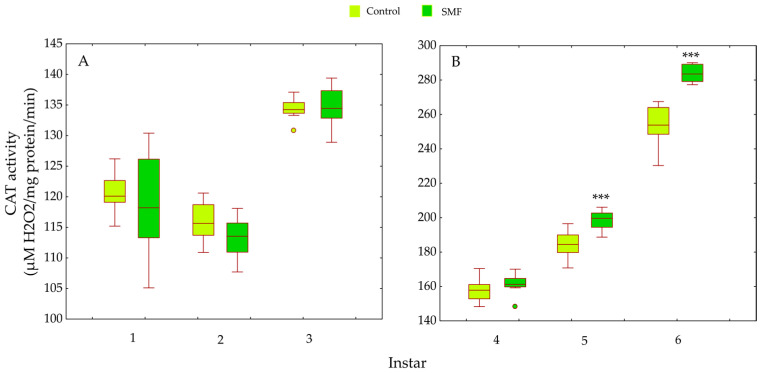
Effect of SMF (110 mT) on catalase (CAT) activity in *L. dispar* larvae across developmental instars: (**A**) first to third instars; (**B**) fourth to sixth instars. Data are presented as boxplots (median, first and third quartiles, minimum and maximum values; *n* = 8 per group). Asterisks indicate significant differences compared to the control group (*** *p* < 0.001; two-way ANOVA followed by Bonferroni post hoc test).

CAT activity was significantly affected by treatment (F_1,84_ = 30.84, *p* < 0.001) and larval instar (F_5,84_ = 1330.93, *p* < 0.001). A significant treatment × instar interaction was also observed (F_5,84_ = 15.82, *p* < 0.001), indicating that the magnitude of the SMF effect varied across developmental stages. In the first and second instars, SMF exposure resulted in decreased CAT activity; however, these differences were not statistically significant. In the third instar, CAT activity was comparable between groups (Figure 6A). A slight increase was observed in the fourth instar, whereas significantly higher CAT activity was recorded in the fifth and sixth instars (*** *p* < 0.001; Figure 6B).

**Figure 7:**
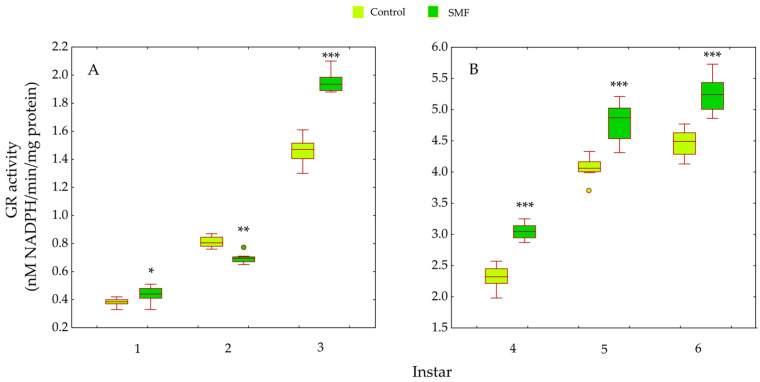
Effect of SMF (110 mT) on glutathione reductase (GR) activity in *L. dispar* larvae across developmental instars: (**A**) first to third instars; (**B**) fourth to sixth instars. Data are presented as boxplots (median, first and third quartiles, minimum and maximum values; *n* = 8 per group). Asterisks indicate significant differences compared to the control group (* *p* < 0.05, ** *p* < 0.001 and *** *p* < 0.001; two-way ANOVA followed by Bonferroni post hoc test).

For GR activity, significant main effects of treatment (F_1,84_ = 106.60, *p* < 0.001) and larval instar (F_5,84_ = 3338.43, *p* < 0.001) were detected, along with a significant treatment × instar interaction (F_5,84_ = 21.99, *p* < 0.001). No consistent trend in GR activity was observed among younger larvae. In the first and third instars, SMF-exposed larvae showed significantly higher GR activity than controls (* *p* < 0.05 and *** *p* < 0.001, respectively), whereas in the second instar, GR activity was significantly lower in the SMF group (* *p* < 0.05; Figure 7A). In contrast, a marked increase in GR activity was observed in SMF-treated larvae during the fourth to sixth instars (*** *p* < 0.001; Figure 7B).

**Figure 8:**
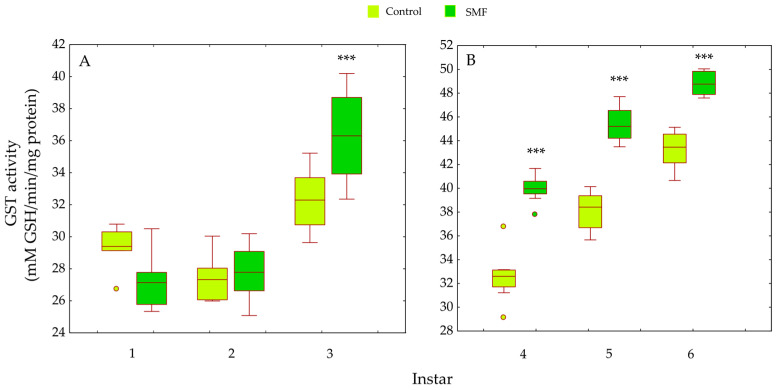
Effect of SMF (110 mT) on glutathione-S-transferase (GST) activity in *L. dispar* larvae across developmental instars: (**A**) first to third instars; (**B**) fourth to sixth instars. Data are presented as boxplots (median, first and third quartiles, minimum and maximum values; *n* = 8 per group). Asterisks indicate significant differences compared to the control group (*** *p* < 0.001; two-way ANOVA followed by Bonferroni post hoc test).

GST activity was significantly influenced by treatment (F_1,84_ = 113.56, *p* < 0.001), larval instar (F_5,84_ = 290.19, *p* < 0.001), and their interaction (F_5,84_ = 20.63, *p* < 0.001), reflecting differential responses across developmental stages. Compared to the control group, SMF exposure significantly increased GST activity in third instar larvae (*** *p* < 0.001; Figure 8A) and in all older instars (*** *p* < 0.001; Figure 8B).

**Figure 9:**
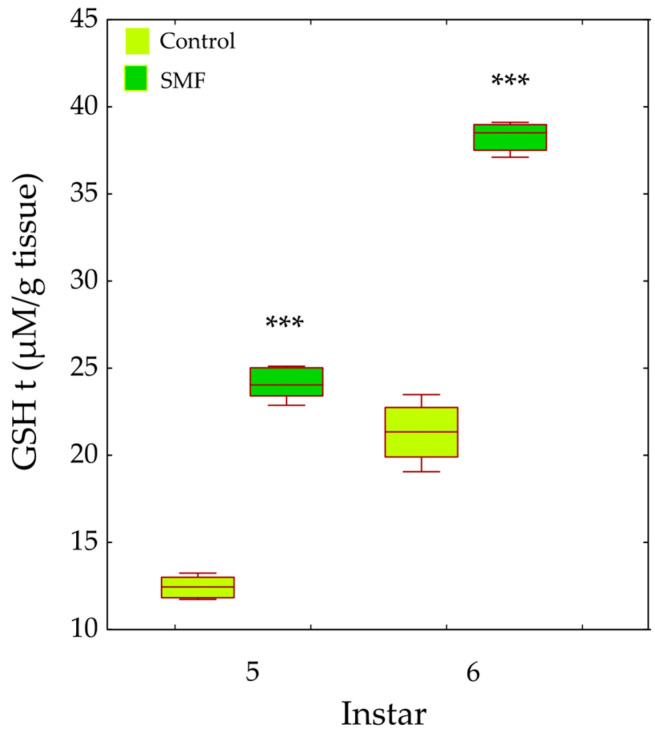
Effect of SMF (110 mT) on total glutathione (GSHt) content in the fifth and sixth larval instars of *L. dispar*. Data are presented as boxplots (median, first and third quartiles, minimum and maximum values; *n* = 8 per group). Asterisks indicate significant differences compared to the control group (*** *p* < 0.001; two-way ANOVA followed by Bonferroni post hoc test).

The total glutathione (GSHt) content in the fifth and sixth instars was significantly affected by treatment (F_1,28_ = 154.96, *p* < 0.001) and by instar (F_1,28_ = 69.37, *p* < 0.001). GSHt content was significantly higher in the SMF group than in the control group in both the fifth and sixth instars (*** *p* < 0.001; Figure 9).

**Figure 10:**
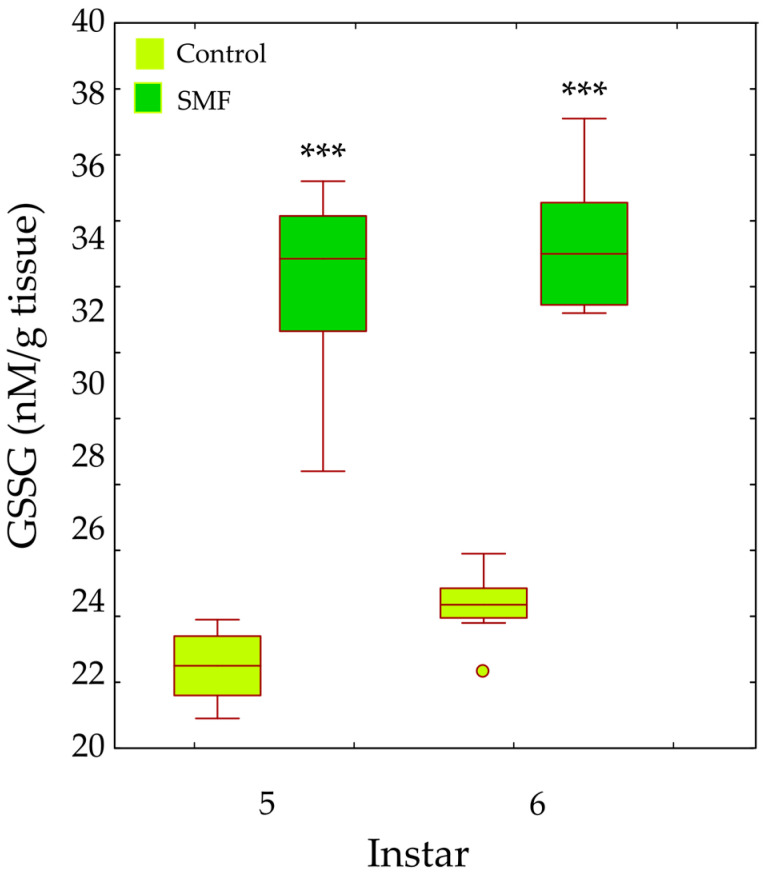
Effect of SMF (110 mT) on oxidised glutathione (GSSG) content in the fifth and sixth larval instars of *L. dispar*. Data are presented as boxplots (median, first and third quartiles, minimum and maximum values; *n* = 8 per group). Asterisks indicate significant differences compared to the control group (*** *p* < 0.001; two-way ANOVA followed by Bonferroni post hoc test).

Two-way ANOVA revealed significant main effects of treatment (F_1,28_ = 274.85, *p* < 0.001) and larval instar (F_1,28_ = 4.88, *p* = 0.005) on oxidised glutathione (GSSG) content in the fifth and sixth instars. GSSG levels were significantly higher in SMF-exposed larvae than in controls in both instars (*** *p* < 0.001; Figure 10).

## 4. Discussion

The effects of magnetic fields on the preadult development of insects, including Lepidoptera, remain poorly understood. A better understanding the wide-ranging effects of magnetic fields during the preadult developmental stage, the most sensitive phase of their life cycle, is needed. The results of our work contribute to clarifying this issue. The differences in the response of younger and older larvae and pupae to magnetic fields, as demonstrated in this study, are particularly important. Clarifying preadult stress sensitivity could improve approaches to environmental risk assessment of various stressors [33,34,35,36,37,38,39] and the development of pest control strategies based on the sensitivity of certain larval stages to the combined effect of magnetic fields and insecticides [40].

### 4.1. SMF Influences on Life History Traits of Pre-Adult Stages of L. dispar

#### 4.1.1. Effects of SMF on Larval and Pupal Survival and Mass

Our results show that the response of *L. dispar* larvae to SMF (110 mT) mainly depends on their developmental stage. On the other hand, the larval stage does not significantly affect mortality, and estimated mortality is higher in larvae exposed to SMF than in control larvae. SMF has no significant effect on the mass of younger larvae, while the mass of older larvae and pupae exposed to SMF is lower than that of the control group (Table 1). As the larvae mature, they accumulate more and more energy reserves, especially in the form of lipids and proteins [41,42], which increases their ability to cope with stress. Prolonged stress is a challenge for insects, as it requires a balance of energy distribution between maintaining essential physiological functions and activating energy-intensive defence mechanisms. Under such conditions, insects use most of their metabolic energy for stress management processes to ensure their survival. It is known that the larvae of *L. dispar*, as well as those of other species, can lose mass under stress due to increased energy consumption [33,34,35,43,44]. Magnetic fields influence the mass and mortality of larvae and pupae in different ways. For example, the fat body mass of one-month-old *Blaptica dubia* nymphs exposed to SMF (110 mT) for five months was significantly reduced [1]. Body weight and growth rate were also affected by magnetic fields in larvae of *Spodoptera littoralis* (~0.218 mT), *Rhynchophorus ferrugineus* (~2.49 mT), and *Galleria mellonella* (~8.63 mT) [45]. Interestingly, exposure of younger *Corcyra cephalonica* larvae to a magnetic field for 12 h had a positive effect on larval weight, while exposures of 0.5 h, 1 h, or 24 h negatively affected larval growth and development [46]. An electromagnetic field (7 mT, 50 Hz) was also shown to increase mass loss in *Tenebrio molitor* larvae [3]. Similarly, increased weight loss was observed in young rats exposed to a pulsed magnetic field (0.1 mT, 60 Hz) [47]. In terms of mortality, SMF is known to increase the mortality rate of larvae of *Corcira cephalonica* (Lepidoptera) eight days after exposure (younger larvae) compared to the control, with the magnitude of this effect depending on the duration of exposure [46]. Similar results were reported in *Culex pipiens* larvae, whose mortality under SMF (5, 25 and 50 mT) was significantly higher than in the controls [48]. Exposure of Drosophila eggs to SMF (4.5 mT) also increased larval mortality [49]. The higher mortality observed in the SMF-exposed group indicates increased sensitivity to magnetic field-induced stress. Early instars are generally more vulnerable due to underdeveloped detoxification capacity and immature antioxidant defences, while cumulative physiological and oxidative stress may contribute to increased mortality in later developmental stages [50]. The elevated mortality recorded following exposure to SMF (110 mT) in our study is consistent with stage-dependent susceptibility and the progressive accumulation of oxidative stress.

#### 4.1.2. Effects of SMF on the Duration of Larval and Pupal Development

The prolonged duration of larval and pupal development after exposure to SMF (110 mT) in our study could be a consequence of the reduced mass. Under stress conditions, a redistribution of energy resources takes place in the larvae. To compensate for the mass loss, they may extend the feeding time to accumulate sufficient reserves and reach the “critical larval mass” required for a successful transition to the next instar [51]. The weight loss may act as a signal to the neuroendocrine system and trigger the synthesis of important developmental hormones in insects such as juvenile hormone (JH) and ecdysone [52,53]. Repeated larval moults are induced by ecdysteroid in the presence of JH, while metamorphosis begins after several moults [54]. JH prevents premature metamorphosis and allows multiple moults until the larva reaches an appropriate size [55], thus acting in contrast to ecdysone in regulating the developmental trajectory and growth duration of insects [56]. The absence of JH at a critical point near the end of larval development enables moulting to the pupal stage. Depending on the developmental stage (larva, pupa or adult), successive high-level cellular signalling pathways are activated, recruiting different downstream gene sets that differ quantitatively rather than qualitatively in their expression [57]. Under stress, JH levels can remain elevated over a prolonged period of time [58,59]. Our previous studies have also shown that the neuroendocrine system of insects is involved in their response to magnetic field exposure as a stressor. The morphometric characteristics of protocerebral A1 and A2 neurosecretory neurons was altered in pupae of the yellow mealworm *T. molitor* exposed to an SMF of 320 mT [60]. In addition, the size of protocerebral A1’ and L2’ neurosecretory neurons of *L. dispar* larvae (fourth instar) was altered at 235 mT, leading to a change in their activity. These neurons synthesise bombyxin, a neurohormone that plays a key role in the regulation of growth, mass, energy metabolism and homeostasis [61]. During the feeding period, energy reserves (lipids, glycogen and proteins) accumulate in the last larval stage, and pupal mass is directly related to larval mass. However, when pupae are exposed to stress factors, including SMF, these energy reserves can be depleted and they lose mass. In our study, the mass of pupae of *L. dispar* (both sexes) under SMF (110 mT) was significantly lower compared to the mass of control individuals (Figure 1). This result is to be expected as the pupae do not feed and the last larval stage already showed a lower mass after SMF exposure. In addition, prolonged larval development leads to the formation of smaller pupae [62]. Under stress conditions, energy allocation shifts from growth to maintenance and defence, particularly towards the activation of antioxidant systems [63,64]. This reallocation constrains somatic growth and developmental progression, explaining the reduced pupal mass and prolonged development observed in the SMF-exposed group. Sex-specific differences in pupal mass may also reflect differences in pupation thresholds and growth ratios [65].

Previous studies show that SMF alters processes that affect fitness-related traits in insects at different life stages [66,67]. These effects are often contradictory and depend on the type and characteristics of the applied magnetic field [68,69]. SMF is known to prolong larval and pupal development in the oriental armyworm *Mythimna separata* [70], while near-zero magnetic fields slow down egg and nymph development in *Laodelphax striatellus* and *Nilaparvata lugens* [71]. The total lifespan of larvae and pupae of the black cutworm *Agrotis ipsilon* increased after exposure to an SMF of 180 mT for 60 min [72]. Populations of *D. subobscura* from oak and beech habitats showed prolonged development after exposure to a strong static magnetic field (SMF) of 2.4 T [73]. Conversely, exposure to SMF (mT) shortened developmental time in *Baculum extradentatum* [74] and in *D. melanogaster* and *D. hydei*, whose eggs were exposed to SMF (60 mT) [67]. Biological parameters such as larval weight, length, and mortality were negatively affected after 4 h magnetic field exposure during rearing of rice moth larvae *Corcyra cephalonica* [75]. The overall duration of the life cycle of insect can be prolonged under MF due to the effects on proteins and enzymes that are activated at certain stages of development [72].

### 4.2. Enzymatic and Glutathione Responses

Considering that static magnetic fields cause oxidative stress through the production of free radicals [76], our research focused on antioxidant protection during the preadult developmental stage of *L. dispar*, a phase that is crucial for minimising oxidative damage. The antioxidant defence system is highly conserved throughout the animal kingdom [77,78] and relies on energetically demanding mechanisms that require significant energy resources, including the mobilisation of energy-rich molecules such as trehalose and glucose, and the production of ATP, NADPH, etc. [79,80,81,82]. This system is crucial for the survival of insects under stress conditions, as it enables a rapid reduction in ROS levels and helps protect cellular components/processes from the damage they can cause—such as changes in the cell membrane; damage and destruction of mitochondrial components, DNA and lipids; and oxidation of amino acids in proteins, leading to changes in their structure. These rapid or early responses of the antioxidant system are critical for the maintenance of cellular integrity and essential for the survival of the organism [83]. Studies on larvae of *L. dispar* confirm that the constitutive activity of certain antioxidant defence enzymes increases with larval maturation [84]. In the larvae of the non-mulberry silkworm *Antheraea militta*, SOD activity also increases during larval metamorphosis from the fourth to the fifth instar [85]. In the fifth instar of the silkworm *Bombyx mori*, the activities of catalase and ascorbate peroxidase also increased over time [86].

### 4.3. Effects of SMF on Enzymatic and Glutathione Responses in L. dispar Larvae

#### 4.3.1. Enzymatic Responses

Our results show that the antioxidant defence strategy depends on the developmental stage (Figure 5, Figure 6, Figure 7, Figure 8, Figure 9 and Figure 10) and is crucial for the maintenance of normal development [85]. In the youngest larvae (first and second instars), SMF does not cause significant changes in the activity of the protective antioxidant enzymes—SOD, CAT, and GST (Figure 5, Figure 6 and Figure 8). Studies have shown that certain protective molecules are transferred maternally to the eggs of *L. dispar*, and may provide protection during the early larval instars [87]. Vitellogenin in insect eggs not only serves as a food source but also contributes to antioxidant defence [88,89], and thus, protects young larvae during early development. In *Apis mellifera*, vitellogenin plays a dual role in both immunity and oxidative stress response, protecting developing larvae from oxidative damage. It participates in the binding and neutralisation of reactive oxygen species (ROS), and thus, represents a form of protection against oxidative stress [90]. In addition to the antioxidant stress system, some heat shock proteins in insects act as molecular chaperones, that preserve the structure of proteins in cells exposed to certain stress factors [91]. The younger larval stages feed on young leaves and buds (preferably of oak species), which contain various bioactive compounds known to help neutralise free radicals and reduce oxidative stress, such as phenolic compounds, flavonoids, carotenoids, tannins, and proline [92,93].

The last (third) younger larval instar reacted to SMF in a similar way to the older instars (fourth to sixth): compared to controls, all analysed antioxidant defence parameters (both enzymatic and non-enzymatic) were increased after exposure to SMF (Figure 5, Figure 6, Figure 7 and Figure 8). As mentioned above, SMF induces the production of ROS, which triggers increased SOD activity. This, in turn, leads to excessive production of H_2_O_2_ within the cells. As this molecule is potentially toxic, increased CAT activity is required to neutralise it and prevent damage to cell structures. This synergistic adaptation mechanism involving SOD and CAT, which represent the first line of antioxidant defence, allows cells to respond effectively to increased oxidative stress caused by the magnetic field [94,95]. The prooxidant effect of static magnetic fields can also act directly on genetic regulators that control the expression of enzymes such as SOD and CAT. For example, a magnetic field can affect the transcription of the genes encoding these enzymes, thereby increasing their production to protect cells from oxidative stress [96,97]. Although CAT activity has a high Km value for H_2_O_2_, excessive accumulation of this molecule may eventually inhibit its activity so that other enzymes such as glutathione S-transferase and ascorbate peroxidase [98,99] must be called upon to remove the peroxide. In *A. mellifera*, the correlations between SOD, CAT, and peroxidase-like enzymes suggest a rational strategy for peroxide management. The increase/decrease in antioxidants must correspond to the concentration and rate of peroxide production [100], a principle that was also confirmed in our previous studies. SOD and CAT activity was increased in *B. extradentatum* reared under SMF (50 mT) compared to control [74]. Similarly, SMF exposure (2.4 T) induced oxidative stress and altered antioxidant defence in *D. subopscura* from oak forests: SOD and CAT activity increased, while glutathione levels remained unchanged [73].

#### 4.3.2. Glutathione System Responses

In younger larval instars, our study showed that SMF significantly alters the activity of glutathione reductase, suggesting that the magnetic field can affect glutathione status (Figure 7A)—an important intracellular antioxidant and redox regulator. SMF is thought to influence the glutathione cycle, one of the key systems for maintaining redox balance. Glutathione concentration is vital for many biological functions and processes, and the glutathione cycle enables their regeneration, and restores the cell’s ability to eliminate free radicals and reactive oxygen molecules [101]. The glutathione system can therefore respond quickly and adaptively to oxidative stress and offers effective and rapid protection at relatively low energy cost. However, excessive production of reduced glutathione can reduce the efficiency of glutathione reductase. If the GSSG concentration becomes too high (substrate saturation), the efficiency of the enzyme may decrease as it becomes completely bound to the substrate and is no longer able to “process” all the available GSSG. A study on Taenia crassiceps metacestodes showed that increased GSSG levels inhibit enzyme activity due to substrate binding, illustrating how GSSG accumulation can impair the function of glutathione reductase [102].

The increased GR and GST enzyme activities (Figure 7 and Figure 8), as well as the content of total and oxidised glutathione—GSHt and GSSG—in older larval stages (Figure 9 and Figure 10), suggest that these enzymes may play a protective role against SMF-induced oxidative stress. Oxidative stress requires rapid regeneration of GSSG to GSH, which can directly scavenge certain free radicals and ROS, i.e., hydroxyl radicals, lipid peroxyl radicals, hydrogen peroxide, etc. The activity of GR and glutathione content are closely linked and are altered differently by different stressors [103]. GR is a key enzyme responsible for maintaining the cellular reduced GSH pool by catalysing the reduction in GSSG to GSH (a major reducing thiol found in most cells and known for its antioxidant properties), with the concomitant oxidation of NADPH [103,104]. Therefore, GR plays a crucial role in maintaining the normal function of GSH-dependent enzymes [105]. The concentration of GSSG was significantly increased in older larvae exposed to SMF (110 mT), which could explain the increase in GR activity as a compensatory mechanism [101]. The main effect of GR is achieved by recycling GSH and maintaining the GSH/GSSG ratio under stress conditions [103].

In a static magnetic field (110 mT), the increased SOD and GST activity probably leads to a reduction in the content of reducing substances in the cells—one of the most important is GSH, which is responsible for the reduction and functionality of oxidised molecules and can be rapidly consumed, especially in stressed larvae. It is certain that the applied MF induced stress in the larvae of *L. dispar*, which led to a change in the redox balance in the cells. Glutathione S-transferase (GST) is an important detoxification enzyme that catalyses the conjugation between GSH and various xenobiotics [106]. This enzyme utilises GSH as a substrate and maintains the thiol status of the cell [85]. Increased GST activity in larvae under SMF (110 mT) helps to reduce oxidative damage and maintain the redox balance in the cell—GST is one of the enzymes that act as a protective agent against radical damage and oxidative stress [107]. In stressful situations, GSSG increases as more GSH, an important factor in protecting organisms from toxicity [108], is utilised to remove free radicals.

#### 4.3.3. Sex-Specific Differences

The response to SMF was most pronounced in the fifth and sixth larval instars of *L. dispar*, which are the final larval instars of males and females, respectively (Figure 5B, Figure 6B, Figure 7B and Figure 8B). Female larvae, preparing for pupation, accumulate energy primarily as lipids to support metamorphosis and future egg production [109], while male larvae store less fat, reflecting their different reproductive and metabolic requirements. After pupation, the stored lipids are crucial for maintaining metabolic and morphogenetic activity during the pupal stage and for egg production in the adult stage. Therefore, females store energy mainly as lipids to ensure successful reproduction and egg development [110,111]. The increased oxidative stress observed in older female larvae may be linked to NADPH consumption associated with lipid biosynthesis, as lipid accumulation is an energy-intensive process that can generate reactive oxygen species. Magnetic field exposure may further exacerbate oxidative stress by affecting metabolic pathways involved in NADPH utilisation and mitochondrial activity [112,113]. Although there is a biochemical rationale connecting lipogenesis and ROS production, lipid content was not directly measured in this study; therefore, this remains a plausible hypothesis rather than a confirmed mechanism, and future studies could include lipid profiling to test this relationship. SOD, CAT, GR, and GST are key enzymes involved in the elimination of free radicals and toxic molecules generated by their action, and in the maintenance of homeostasis. Antioxidant protection is essential during magnetic field-induced stress in insects, as we have previously showed [26,73,74]. During preadult development of *L. dispar*, the activity of these enzymes increases to counteract increased free radical concentrations. A static magnetic field may act as a factor that modulates this response, possibly enhancing enzyme synthesis or activity, or even promoting interactions between different enzyme systems, thus contributing to improved cell protection [97,114].

The results of this study reflect the acute effects of SMF (110 mT), a field strength that substantially exceeds the geomagnetic background, which chronically influences living organisms. The 48 h exposure window coincides with a critical phase of larval development, characterised by elevated metabolic and physiological activity, making it particularly suitable for detecting acute magnetic stress responses. Under these conditions, larvae showed a coordinated increase in antioxidant enzyme activities and glutathione levels, indicating activation of defence mechanisms aimed at mitigating oxidative damage. However, this response appears insufficient to fully counteract stress effects, as evidenced by reduced growth and increased mortality. The stage-specific pattern observed—initial stimulation followed by detrimental effects—may reflect a hormetic-like response, in which early or short-term exposure induces transient adaptive responses, whereas prolonged or cumulative exposure results in physiological costs [115]. Using 110 mT allows us to investigate larval and pupal sensitivity to magnetic fields well above natural background levels, providing insights into how insects respond to stronger environmental magnetic fluctuations.

In this study, we focused on magnetic fields with specific characteristics and selected physiological and molecular markers. Further research is needed to investigate additional biomarkers of oxidative stress and assess the potential effects of magnetic fields of different durations and characteristics on insect physiology.

## 5. Conclusions

Exposure to static magnetic fields (110 mT) induces significant physiological changes during preadult development of *L. dispar*. The obtained results extend our knowledge of the differences in the effects of magnetic fields on larval mortality, mass, duration of larval and pupal development and antioxidant protection in younger and older larval stages of *L. dispar*. The marked differences in responses observed in all larval stages of *L. dispar* are critical to understanding how magnetic fields, an increasingly prevalent and dangerous environmental stressor, affect the biology and survival of this indicator species of environmental change and an important forest pest. In addition to these fundamental findings, these results may enable the development of more targeted and effective strategies for the management of *L. dispar* population, and for the assessment of the overall impact of magnetic pollution on wildlife.

## Figures and Tables

**Table 1:** Mass of *L. dispar* larvae (mg) under control and SMF treatments. Data are presented as means ± SEM (*n* = 12 pools of 10 larvae for instars 1–2, *n* = 30 larvae for instars 3–6). Differences were analysed using GLM followed by the Bonferroni post hoc test.

Mass (mg)	Instars	1	2	3	4	5	6
	Control	48.0 ± 0.8	294.8 ± 5.3	89.9 ± 0.9	190.1 ± 1.6	368.9 ± 1.6	713.6 ± 4.4
	SMF	48.5 ± 1.3	293.8 ± 4.5	90.4 ± 0.8	153.8 ± 1.9	276.7 ± 3.0	629.2 ± 3.0
	*p*	>0.05	>0.05	>0.05	<0.001	<0.001	<0.001

## Data Availability

The raw data supporting the conclusions of this article will be made available by the authors on request.

## Abbreviations

The following abbreviations are used in this manuscript:

SMF	Static magnetic field
ROS	Reactive oxygen species
SOD	Superoxide dismutase
CAT	Catalase
GR	Glutathione reductase
APOX	Ascorbate peroxidase
GST	Glutathione-S-transferase
GSH	Glutathione GSH
GSHt	Total GSH
GSSG	Oxidised glutathione
